# Topical and Mucoadhesive Administration of Capsaicin in the Burning Mouth Syndrome Treatment

**DOI:** 10.3390/jcm15020780

**Published:** 2026-01-18

**Authors:** Jacek Zborowski, Bożena Karolewicz, Arleta Dołowacka-Jóźwiak, Dawid Bursy, Krzysztof Słotwiński, Tomasz Konopka

**Affiliations:** 1 Department of Periodontology and Oral Pathology, Wroclaw Medical University, 50-367 Wrocław, Poland; 2 Department of Drugs Form Technology, Wroclaw Medical University, 50-367 Wrocław, Poland; 3 Department and Clinic of Neurology, Wroclaw Medical University, 50-367 Wrocław, Poland

**Keywords:** burning mouth syndrome, topical treatment, capsaicin

## Abstract

**Background/Objectives:** Burning Mouth Syndrome (BMS) is a common oral condition in older women and is characterized by a multifactorial etiology. To date, no standardized treatment strategy has been established. The aim of this study was to evaluate the effectiveness of topical application of capsaicin (0.025 mg/cm^2^) in the form of a mucoadhesive bilayer polymer reducing burning sensations in BMS. The study assessed levels of depression, sleep disturbances, and quality of life. **Material and Methods:** The proof-of-concept study included 29 patients with symptoms of BMS. The peripheral origin of BMS was confirmed by lingual nerve block. Pain intensity was assessed using the Numeric Rating Scale (NRS-11) and the Short-Form McGill Pain Questionnaire (SF-MPQ). Depression, sleep disturbances, and quality of life were evaluated using the Beck Depression Inventory (BDI), Athens Insomnia Scale (AIS), and WHO Quality of Life Questionnaire (WHOQoL). **Results:** A reduction in pain was observed in over 86% patients. Decrease in burning at treatment sites was recorded immediately after treatment and also at the 3-month follow-up. Gender, taste disturbances, depression, and age were found to have a significant effect on final NRS-11 scores. **Conclusions:** Significant reduction in pain intensity was achieved in nearly all treated patients, with adverse effects being rare.

## 1. Introduction

Burning Mouth Syndrome (BMS) is currently defined as an intraoral burning sensation or dysesthesia that occurs daily for at least two hours and persists for a minimum of three months, in the absence of clinically evident mucosal lesions [1]. The pain is typically superficial and described as burning, with no identifiable local causative factors on clinical examination and no systemic conditions revealed through available diagnostic tests.

According to the International Classification of Orofacial Pain (ICOP)*,* BMS can be categorized into forms without any qualitative or quantitative alterations in somatosensory testing, and those accompanied by such disturbances, including reduced sensory sensitivity or taste distortions (e.g., dysgeusia) [2].

The primary subjective clinical symptom is most commonly a bilateral burning sensation affecting the anterior two-thirds of the tongue. This may be accompanied by xerostomia without hyposalivation, taste disturbances (such as reduced sensitivity to sweet and salty tastes or phantom tastes like bitter or metallic), impaired sleep quality, and psychiatric comorbidities including anxiety and depression [3].

Depending on the diagnostic criteria applied, the prevalence of BMS in the general population is estimated to range from 0.1% to 3.7%, with a marked predilection for middle-aged and older individuals—particularly peri- and postmenopausal women—among whom the condition is at least seven times more common than in men [3].

In the etiopathogenesis of BMS, one proposed mechanism involves a focal peripheral sensory neuropathy of the lingual nerve and chorda tympani is suggested, accompanied by a significant increase in the expression of transient receptor potential vanilloid 1 (TRPV1), nerve growth factor (NGF)-positive fibers, and the purinergic receptor P2X3 [4,5]. A reduction in mucosal innervation of the tongue, together with an increased expression of TRPV1 nociceptors and NGF-positive nerve fibers, has been described particularly in subsets of patients with BMS, supporting the concept of a peripheral neuropathic phenotype rather than a universal mechanism.

Damage to intraepithelial Aδ afferent taste fibers of the chorda tympani has also been observed, which may lead to reduced inhibitory control over C-fibers responsible for the burning phantom pain widely considered the hallmark of BMS [6].

Other theories have suggested a central neuropathological mechanism for BMS. PET studies have demonstrated impaired function of the nigrostriatal dopaminergic system, which may result in a reduction in endogenous pain inhibition within the dopaminergic–opioid pain modulation pathway of the brain [7]. Alterations in dopamine D2 receptor expression may be linked to a polymorphism of the DRD2/ANKK1 TaqIA (rs1800497) gene (commonly referred to as polymorphism 957). Similarities in neurochemical changes between BMS and Parkinson’s disease have also been reported, particularly involving reduced dopamine release in cortical regions responsible for pain processing [8].

Additionally, BMS has been associated with dysfunction of the endogenous descending pain modulation pathway, potentially leading to central sensitization as a consequence of the ongoing failure of chronic pain inhibition mechanisms [9].

Previous attempts at treating Burning Mouth Syndrome (BMS) have shown limited efficacy, with sustained remission of burning symptoms being rare. The most favorable outcomes have been reported following the topical application of clonazepam. Other pharmacological agents that have been used include various anxiolytics, such as selective serotonin reuptake inhibitors (SSRIs), sertraline, fluoxetine, and citalopram, as well as tricyclic antidepressants (amitriptyline, nortriptyline, doxepin), trazodone, pregabalin, gabapentin, and alpha-lipoic acid [10].

Capsaicin has been administered in the treatment of BMS both systemically [11] and topically [12,13,14,15,16]. Capsaicin is a highly selective agonist of the TRPV1 receptor. Upon topical administration, the initial effect involves activation of TRPV1 nociceptors and membrane depolarization through the opening of calcium channels and subsequent influx of calcium and sodium ions. This ionic movement triggers action potentials in sensory neurons, perceived as burning pain or itching.

Subsequently, particularly at low concentrations of capsaicin, the continued influx of calcium ions leads to the inactivation of sodium and Piezo2 channels, resulting in the blockade of sensory signal transduction and in the defunctionalization and desensitization of hyperreactive pain receptors. This leads to a temporary, weeks-long blockade of C-fiber nerve endings, resulting in a significant reduction in neuropathic pain [17]. In previous attempts at topical treatment of BMS with capsaicin, a 0.02% mouth rinse [12,13,16] or a 0.01–0.025% gel [14,15] was used. The use of a mucoadhesive dressing in the present study allows the action of capsaicin to be limited exclusively to the site of pain and reduces the risk associated with systemic adverse effects.

The aim of this study is to evaluate the medium-term effectiveness in pain reduction following topical application of capsaicin (0.025 mg/cm^2^) in the innovative form of a mucoadhesive bilayer polymer applied to the tongue in reducing burning sensations in patients with BMS, with particular focus on individuals in whom peripheral nociceptive mechanisms are hypothesized to contribute to symptom generation. In addition, indicators of depression, sleep disturbances, and quality of life will be assessed in the study group, and clinical and psychological variables significantly associated with pain reduction after topical capsaicin administration will be identified.

## 2. Materials and Methods

The study included 29 patients from the Department of Periodontology at the Wroclaw Medical University, all diagnosed with Burning Mouth Syndrome (BMS). There are currently four classifications of orofacial pain (ICOP, ICHD-3, IASP, DSM-V) in which BMS is distinguished. In our study, we adopted the ICOP classification, in which secondary BMS does not occur and BMS is diagnosed only if local and systemic causes are excluded. The 2020 definition of BMS according to the International Classification of Orofacial Pain (ICOP) [2] is as follows: an intraoral burning sensation or dysesthesia occurring daily for at least 2 h and persisting for more than 3 months, potentially accompanied by somatosensory disturbances such as xerostomia or taste alterations, with a clinically normal-appearing oral mucosa.

Exclusion criteria included systemic factors potentially causing oral pain or burning: trigeminal or facial neuralgia, SARS-CoV-2 infection, diabetes mellitus, hypothyroidism, anemia, deficiencies of iron, vitamin B12, or folic acid, autoimmune diseases, Parkinson’s disease, salivary gland disorders associated with reduced salivary secretion, systemic treatment with selected drug classes (angiotensin-converting enzyme inhibitors, anxiolytics and antidepressants, hormones and their derivatives, antiviral drugs), and diagnosed peripheral neuropathies (e.g., amyloid, postherpetic, or HIV-related neuropathy).

Local exclusion criteria involved oral conditions capable of mimicking BMS symptoms, including acute or chronic oral candidiasis, geographic tongue, oral lichen planus, denture stomatitis, contact allergic mucositis, mechanical trauma, and parafunctional habits.

All patients underwent the following laboratory tests: complete blood count, fasting glucose, serum levels of iron, folic acid, vitamin B12, TSH, and CRP. In diagnostically ambiguous cases, patients were referred for microbiological culture to rule out candidiasis.

Before enrollment, all participants provided written informed consent to participate in medical experiment, which was approved by the Bioethics Committee of Wroclaw Medical University (approval no. KB 182/2023). The experiment was conducted from March 2024 to September 2025 as part of a university-funded research grant (SUBZ.B050.23.50).

To confirm peripheral sensory neuropathy as the predominant mechanism of BMS, each patient underwent a lingual nerve block with 1 mL of 3% mepivacaine hydrochloride without epinephrine prior to study inclusion. Complete relief or a marked reduction in burning sensation within 10 min after the injection was considered indicative of a peripheral origin of the pain symptoms [18].

After qualifying for the study, the following parameters were assessed (TK): pain/burning intensity using the Numeric Rating Scale (NRS-11) from 0 (no pain) to 10 (worst imaginable pain) [19], a Short-Form McGill Pain Questionnaire (SF-MPQ) with a maximum score of 45 [20], taste disturbances (reduced/distorted perception of specific tastes, perception of tastes unrelated to food consumption), presence of xerostomia, active smoking, number of remaining teeth, and type of prosthetic restoration. Immediately after the clinical assessment, a psychologist (KS) performed psychological tests: Beck Depression Inventory—BDI [21], Athens Insomnia Scale—AIS [22], and patients’ quality of life based on the short version of the WHO Quality of Life Questionnaire (WHOQoL—WHO Quality of Life) [23], which evaluates 4 domains: DOM1—physical (maximum score 35), DOM2—psychological (maximum score 30), DOM3—social (maximum score 15), and DOM4—environmental (maximum score 40).

Fragments of unilaterally coated polymeric dressings measuring 2.5 × 2.5 cm and containing 0.025 mg/cm^2^ of capsaicin were used in the treatment. In the studies, the dressings were applied to the dried surface of the tongue after individual adjustment. The dressings were obtained and their physicochemical properties were evaluated at the Department of Pharmaceutical Technology of the Wroclaw Medical University, in accordance with the description provided below.

Bilayer polymer-film with capsaicin—preparation and evaluation of properties

**Figure 1:**
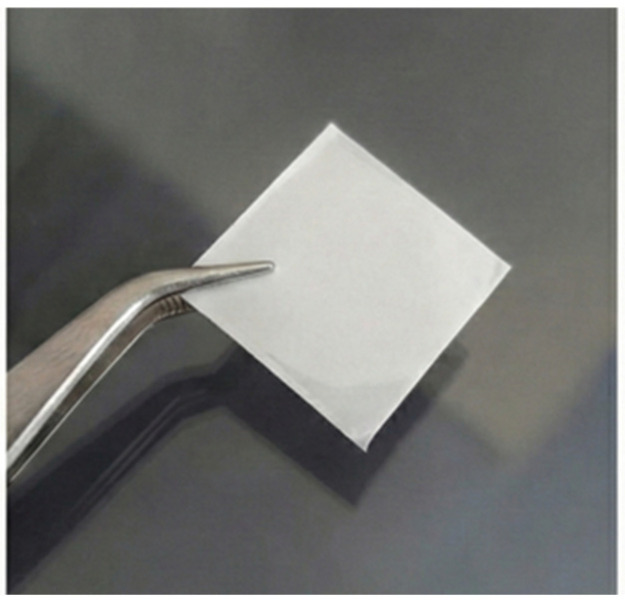
Photograph of a bilayer polymer film loaded with capsaicin (taken with a Nikon S9300 camera using a macro lens).

The bilayer polymer-film was designed utilizing the modified solvent-casting technique [24] to ensure consistent thickness and reliable mechanical performance. The film comprised a backing layer and a mucoadhesive active layer containing the capsaicin (Pol-Aura, Poland, Morąg). The backing layer was prepared by dissolving ethyl cellulose (viscosity 4 cP of 5% in 80/20 toluene/ethanol solution at 25 °C, Sigma-Aldrich, Saint Louis, MO, USA) in solvent mixture consisting of isopropyl alcohol (Sigma-Aldrich, Saint Louis, MO, USA) and acetone (Sigma-Aldrich, Saint Louis, MO, USA). Next, triacetin (Sigma-Aldrich, Saint Louis, MO, USA) as a plasticizer, was subsequently added to the polymer solution. The mixture was agitated in an unguator Eprus^®^ model e/s (Eprus Sp. z o.o., Bielsko-Biala, Poland) at 2340 rpm by 2 min until a homogeneous, viscous gel was obtained and allowed to stand to facilitate the partial evaporation of volatile components and achieve the desired consistency. The resulting wet formulation was cast using an applicator onto a smooth surface up to a thickness of 0.4 mm and dried for 48 h under controlled conditions in a vacuum dryer. The second, mucoadhesive layer was formulated based on hydroxypropyl methylcellulose (viscosity 40–60 cP of 2% aqueous solution at 20 °C, Sigma-Aldrich, Saint Louis, MO, USA) dispersed in co-solvent system of ethanol 96% *v*/*v* (Honeywell, Charlotte, NC, USA) and glycerol 85% (Fagron, Kraków, Poland) with the addition of sorbitol (70% m/m aqueous solution, Sigma-Aldrich, Saint Louis, MO, USA). To ensure complete polymer hydration and homogenization in solvent the mixture was heated in an autoclave at 80 °C for 60 min. Immediately after thermal treatment the hot gel was degassed under vacuum to eliminate air bubbles that could compromise the structural integrity of the film. Upon gel cooling, the capsaicin was incorporated by mixing 10 g of the base gel with 1.5 mL of an ethanolic capsaicin solution (1 mg/mL) to ensure uniform drug content. Next, the gel containing the active substance was covered with a protective liner and spread to a thickness of 0.3 mm, then left to dry. Both layers were joined after drying by heating the protective layer and placing the mucoadhesive layer on top of it, which completed the process of manufacturing the two-layer system. The publication does not specify the quantitative composition due to the ongoing process of preparing a patent application. The bilayer polymer-film after drying is depicted in Figure 1.

The obtained bilayer film formulations were measured at multiple random points using a Hard Head (USA) screw micrometer gauge, reaching a thickness of 0.71 ± 0.06 mm for the capsaicin-loaded mucoadhesive active layer (0.32 ± 0.03 mm) and inert backing layer (0.41 ± 0.02 mm), respectively, which exhibited consistent morphological and structural properties. A thorough visual inspection was conducted, revealing a high degree of uniformity across the entire surface area. No evidence of polymer agglomerates, undissolved particles, or entrapped air bubbles was found within the active matrix or backing coating. The films exhibited desirable mechanical characteristics suitable for application to the oral mucosa: they were flexible, had a smooth texture, and were non-adherent, allowing them to be easily removed after application without causing damage to the backing layer. It is crucial to note that the interface between the backing layer and the mucoadhesive layer exhibited strong cohesion, with no indications of delamination or separation. Mechanical property testing for 2.5 cm × 2.5 cm squares film samples was analyzed and folded back and forth along a fixed line at an angle of 180°. In the flexural strength test to evaluate the mechanical resistance of the bilayer systems tested, it demonstrated excellent flexibility. The bilayer film formulations successfully withstood the maximum flexural threshold of 300 foldings without showing any macroscopic signs of damage, such as cracks or tears.

**Figure 2:**
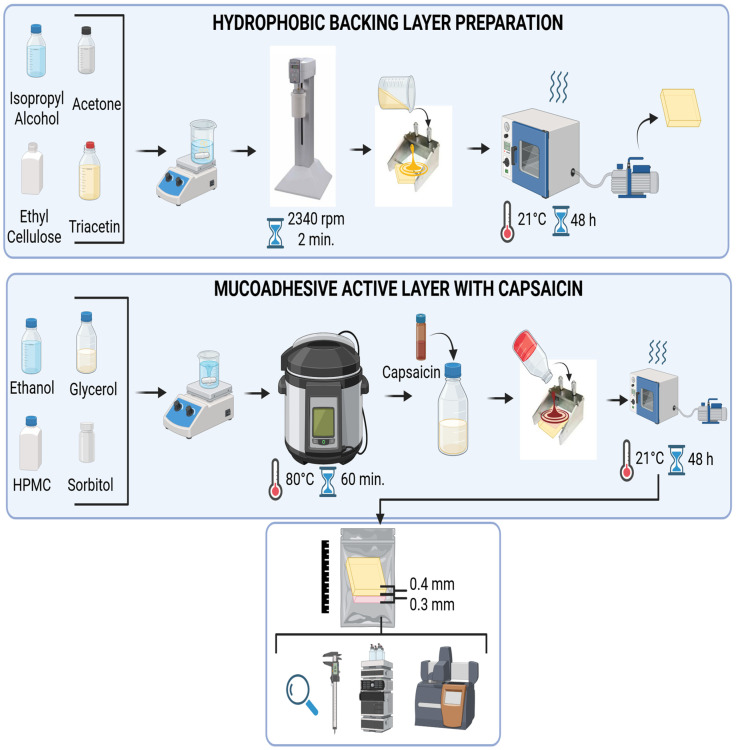
The process of preparing a mucoadhesive carrier containing capsaicin.

To determine the capsaicin content the 2.5 × 2.5 cm film-samples were securely placed in a volumetric dark glass flasks of 25 mL and filled with 20 mL methanol (Sigma-Aldrich, Saint Louis, MO, USA). The flasks were shaken at 200 rpm at room temperature for 30 min and placed in an ultrasonic water bath at 40 °C for another 30 min. The samples were then conditioned at 20 °C for 60 min to sediment the polymer fragments and topped up with methanol to 20 mL. The solution was transferred into amber glass HPLC vials and analyzed using HPLC Agilent 1260 Infinity with UV-DAD detection in view against a calibration curve prepared using standard solutions of three capsaicinoids [25]. The HPLC method developed by Kuzma et al. [25] was performed using a Supelco Ascentis Express C18 analytical column with a diameter of 2.6 μm (100 mm × 4.6 mm) and maintained at a temperature of 25 °C. The mobile phase consisting of a 1:1 mixture of acetonitrile (Sigma-Aldrich, Saint Louis, MO, USA) and 1% *v*/*v* solution of phosphoric acid in water (Sigma-Aldrich, Saint Louis, MO, USA). The analysis was performed through isocratic program at the flow rate 1 mL/min with 10 μL injection volume and detection wavelength at 281 nm. Under the mobile phase conditions, elution of analyte was completed in less than 5 min and retention times of 3.05 min for nordihydrocapsaicin, 3.27 min for capsaicin, and 4.60 min for dihydrocapsaicin. The method was checked for linearity, precision, and specificity against placebo film analysis. The mean sum of capsaicinoids was determined in samples (*n* = 4) at 321.9 ± 19.1 μg, with the calculated content of capsaicin being 167.5 ± 10.5 μg, representing 107.2% of the declared value. Residues of volatile solvents and water in film samples were examined using a Netzsch TG 209 F1 Libra thermogravimetric analyzer, with an applied heating rate of 10 K min^−1^ and a N_2_ gas flow of 50 mL min^−1^. In the temperature range of 25–100 °C, the mucoadhesive layer showed a weight loss of 1.08%, backing layer of 0.75%, and complete two-layer system of 1.22% (Figure 2).

A medical experiment was conducted in patients with bilateral symptoms affecting the dorsal surface of the tongue, and in some cases, additional intraoral sites of burning (such as the anterior palate, mandibular alveolar ridge, or lower oral vestibule).

In cases where symptoms were limited to the tongue, the patch was applied unilaterally with simple randomization (odd-numbered patients received the patch on the left side, even-numbered patients on the right side), with the opposite side of the tongue serving as the control. In cases where burning extended to other intraoral locations, the patch was applied to the entire affected area of the tongue, and a different intraoral site served as the control.

Patch fitting and distribution to patients were carried out by a second clinical investigator (JZ). The patch was applied twice daily (morning and evening) for 10 min over a period of 2 weeks.

Follow-up visits were scheduled after 7 days of treatment, immediately upon completion of the 2-week treatment period, and again at 12 weeks post-treatment. During the visits conducted immediately after treatment and at the final follow-up visit, the physician (TK, blinded to the sites of capsaicin application) assessed pain intensity using the NRS-11 and SF-MPQ scales on both sides of the tongue and at other sites of burning sensation.

To assess the normality of variable distribution, the Kolmogorov–Smirnov test with Lilliefors correction was used. Levene’s test was applied to evaluate the homogeneity of variances between two or more variables. Given the normal distribution and homogeneity of variance, Student’s *t*-test was used for statistical analysis of differences between independent and dependent variables. Spearman’s rank correlation test was applied for correlation analysis.

One-way ANOVA was used to compare three means, and Tukey’s post hoc test was applied for equal sample sizes. To evaluate the influence of selected variables on changes in NRS-11 scores after treatment, a multiple regression model was constructed.

The model was evaluated based on the significance of the linear regression, the significance of the partial regression coefficients, the absence of multicollinearity among the independent variables, the presence of homoscedasticity and the absence of autocorrelation of residuals (Durbin–Watson test), the normal distribution of residuals, and a zero mean of the random error term (ε_i_). For each statistical test except correlation analysis, a *p*-value < 0.05 was considered statistically significant, while in the analysis of covariance, the significance threshold was set at *p* < 0.02. Statistical analysis was performed using Statistica software, version 13.3 (TIBCO Software Inc., Palo Alto, CA, USA).

## 3. Results

The study was completed by 29 patients (22 women and 7 men) aged between 41 and 73 years (mean 61.8 ± 8.3). One patient discontinued treatment during the first week due to a significant increase in blood pressure. The mean baseline NRS-11 value for the treated sites was 6.44 ± 1.6, while for the control sites it was 6.24 ± 1.6. The mean baseline SF-MPQ value for the treated sites was 35.93 ± 7.8, and for the control sites it was 34.2 ± 8.1. Xerostomia without hyposalivation was found in 14 patients (48.3%), taste disturbances were reported by 13 patients (44.8%), and 8 patients (27.6%) were active smokers. Beck Depression Inventory values ranged from 4 to 41 (mean 19.31 ± 10.7), with normal scores (<20) found in 17 patients, moderate depression indicated in 3 patients, and severe depression (>25) in 9 patients. Athens Insomnia Scale values ranged between 2 and 17 (mean 8.51 ± 4.2), with sleep disorders (score ≥ 8) observed in 16 patients (55.2%). The mean and median values of the WHOQoL domains were as follows: physical (DOM-1) 21.07 ± 2.7 and 21, psychological (DOM-2) 19 ± 2.8 and 19, social (DOM-3) 9.17 ± 2.3 and 9, and environmental (DOM-4) 28.69 ± 3.7 and 30. A reduction in pain intensity measured with the NRS-11 was reported by 25 patients (86.2%) immediately after treatment and by 27 patients (93.1%) after 3 months. According to the SF-MPQ, this effect was observed in 28 patients (96.4%) at both follow-up visits.

Before the initiation of treatment, significant positive correlations were found between both indicators of burning intensity and BDI scores (Table 1); these associations were no longer present three months after the completion of therapy.

For both pain intensity indicators, a significant reduction was observed at the capsaicin application sites between baseline and three months after treatment completion. At the final follow-up, a significantly greater reduction in burning sensation, as assessed by both indicators, was maintained at the treated sites compared to the control sites (Table 2).

For both pain intensity indicators, one-way ANOVA revealed a significant decrease in burning sensation at the treated sites not only immediately after the completion of capsaicin application, but also at the three-month follow-up compared to the post-treatment time point (Table 3).

A multiple regression model was constructed in which NRS-11 scores at the end of the 3-month follow-up were significantly associated with age, sex (1—female, 2—male), presence of taste disturbances, and Beck Depression Inventory (BDI) score (F = 8.14, R = 0.76, R^2^ = 0.57, adjusted R^2^ = 0.51, *p* = 0.0002).

During model validation, the following were confirmed: significance of partial regression coefficients (sex—*p* = 0.004, age—*p* = 0.016, taste disturbance—*p* = 0.014, BDI—*p* = 0.007); absence of multicollinearity among independent variables (tolerance and R^2^ values were as follows: for sex 0.90 and 0.10; for age 0.88 and 0.11; for taste disturbance 0.75 and 0.24; for BDI 0.82 and 0.17); the residuals vs. predicted values plot showed a uniform scatter; no significant autocorrelation of residuals was observed (Durbin-Watson statistic = 1.58); residuals were normally distributed; and the mean value of outlier residuals was −2.87. The multiple regression model was as follows:

NRS-11 score at follow-up = 4.36 + 1.68 × sex + 1.31 × taste disturbance + 0.07 × BDI—0.07 × age ± 1.72

## 4. Discussion

The conducted study observed a reduction in the primary clinical symptom of BMS, burning sensation, following the application of individually adapted mucoadhesive patches with capsaicin (0.025 mg/cm^2^) to the site of pain. This finding was also supported by the use of the Short-Form McGill Pain Questionnaire, which assesses not only the sensory dimension of pain but also its affective component (the last four descriptors in the questionnaire), and is applicable in the evaluation of neuropathic pain [26]. A reduction in pain intensity, although not its complete resolution, was observed in more than 86% of patients with BMS undergoing treatment. These outcomes were more favorable than those reported by other authors, namely 67% [12] and 50% [16]; however, direct comparison is limited by differences in study design, control conditions, and treatment protocols. A reduction in oral neuropathic pain after four weeks of capsaicin use (0.025 mg/cm^2^) was reported in 63% of patients [17]. These differences may have resulted from the more localized effect of the mucoadhesive patch at the site of pain in the oral cavity compared to the broader action of capsaicin mouthwash. Another contributing factor could be the more precise selection of patients and the initiation of BMS treatment only after confirming the peripheral origin of pain through lingual nerve block. Even in cases of BMS associated with small fiber peripheral neuropathy, not all patients respond with a significant reduction in pain to treatment with TRPV1 receptor agonists due to polymorphisms in the TRPV1 gene [27]. Table 4 presents a comparison of the extent of burning reduction assessed using the NRS-11 scale after topical application of capsaicin by other authors [12,13,14,15,16] in relation to the present study.

Notable differences in treatment protocols can be observed: the form of capsaicin administration (mouthwash, gel, mucoadhesive patch), study control (placebo, untreated burning areas, boric acid, other agents used in BMS treatment: alpha-lipoic acid, B vitamin complex, zinc), duration of treatment (ranging from 1 to 12 weeks), and follow-up period (none, from 2 weeks to 4 months). Given the short duration of capsaicin use in our study, the observed reduction in burning sensation can be considered highly favorable. At the end of the treatment period, the results were most comparable to those reported by Silvestre et al. [13]. Furthermore, we observed a continued significant decrease in burning sensation during the three-month follow-up, which aligns with the findings of Ricken et al. [15], who reported similar outcomes four months post-treatment. Topical application of low concentrations of capsaicin (0.025–0.075%) in chronic neuropathic pain produces an analgesic effect around day 14 of treatment, and this effect, along with an increased threshold for heat and touch perception, persists for up to approximately six weeks after discontinuation of the drug [17]. The recurrence of neuropathic pain following capsaicin treatment is thought to reflect the capsaicin-induced regeneration of afferent small nerve fibers expressing TRPV1 receptors [17]. The decrease in burning intensity assessed by NRS-11 and described by Ricken et al. [15], from 72% at the end of active treatment to 88% at the 4-month follow-up, may have been partly due to the 12-week application of 0.025% capsaicin gel. In our observation, the reduction was smaller (from 44.9% to 64.8% at the final evaluation) and may have been influenced by a sustained placebo effect. In evaluating the effectiveness of therapies for conditions such as BMS, the placebo effect is a significant confounding factor [14,28]. This is due to the influence of multiple psychological mechanisms. These include the expectation of effective treatment developed over many years of chronic pain; conditioning through frequent follow-up visits and the attention received from multiple specialists; enhanced understanding of BMS; increased focus on describing subjective sensations; the rewarding experience of pain relief; reduction in anxiety; and the feeling of being distinguished by receiving an innovative form of therapy. Control sites with burning sensation that did not receive topical treatment in our observation may be considered analogous to a placebo condition; at the final assessment, the placebo effect was 38.1% based on NRS-11 scores and 33.8% based on SF-MPQ scores. In a systematic review of the literature on the placebo effect in six studies on the treatment of BMS conducted between 1999 and 2012, the magnitude of the placebo effect was estimated to range from 15% to 74%, accounting for up to 72% of the response to pharmacological treatment [29]. In a contemporary systematic review by Rossetti et al. [30] on BMS treatment, the placebo effect in pain reduction was estimated at 20–40%, reflecting the importance of psychological factors in these patients. In addition to psychophysiological and behavioral factors and the use of receptor agonists in treatment, the development of a placebo response in neuropathic pain reduction has also been attributed to endogenous opioid mechanisms that inhibit nociceptive activity [31]. In the network meta-analysis by Alvarenga-Branta et al. [32], it was demonstrated that the only treatment for BMS providing a significantly greater reduction in pain compared to placebo was clonazepam, a medium-acting benzodiazepine and gamma aminobutyric acid (GABAA) receptor agonist, administered topically and/or systemically. Other treatment modalities did not show a significant difference and included photobiomodulation therapy, tongue protectors and phytotherapy, pregabalin, antidepressants, and oral lubricants. However, clonazepam has well-known adverse effects, particularly the potential for dependence, which makes long-term use problematic in elderly patients. In the present study, at the final follow-up, a greater reduction in pain was observed after topical application of the mucoadhesive patch containing capsaicin compared to BMS sites treated as placebo (64.8% vs. 38.1% according to NRS-11, and 71.3% vs. 33.8% according to SF-MPQ). Capsaicin should not be used systemically in BMS due to its adverse effects, such as abdominal pain, vomiting, hypertension, bronchitis, and sinusitis [11]. When applied topically at concentrations of 0.025–0.075%, capsaicin may cause local reactions, particularly at the beginning of treatment, including burning, erythema, and itching at the application site, as well as coughing and sneezing [33]. In our study, treatment was discontinued by only one patient (3.4%) due to hypertension. In four comparable studies, the rates of treatment discontinuation due to topical capsaicin were considerably higher: 44.4% (reasons included allergic reactions and lip erythema) [15], 35.7% (no reasons specified) [12], 23.3% (symptom exacerbation, lack of treatment effect) [13], and 18.2% (gastrointestinal symptoms, throat dryness, unsuitable dosage form) [14]. The very low rate of treatment discontinuation in the present observation indicates the appropriate form of administered capsaicin, and the use of a unilaterally laminated patch, introduced following pilot trials, contributed to the practical absence of local adverse effects. The frequent co-occurrence of oral symptoms such as xerostomia and taste disturbances was confirmed in the patients treated in our study. The prevalence of xerostomia without hyposalivation was consistent with the current literature data (ranging from 46% to 67%), whereas the frequency of taste disturbances was lower, as other authors report that it may reach up to 70% in BMS cases [3]. It has been shown that topical application of 0.025% capsaicin gel to the gingiva for two weeks does not alter the sensory symptom of objective taste function, although a temporary increase in the perception of sweet and salty tastes was observed in some patients [34]. The prevalence of depression based on BDI scores in our group was 41.4%, which falls within the range reported in recent studies—30.8% in Korean patients [35] and 50% in American patients [36]. We confirmed a significant association between the intensity of the burning symptom and the severity of depression. Based on AIS results, insomnia was identified in 55.2% of our patients, although it did not influence the subjective perception of burning. Other studies have reported rather a reduction in sleep quality and efficiency [37]. As a chronic pain syndrome, BMS significantly reduced our patients’ quality of life, with strong correlations observed between decreased scores across all WHOQoL domains and the severity of both sensory and affective subjective symptoms. The network meta-analysis did not demonstrate that any of the treatment methods used to date for BMS improved patients’ quality of life to a greater extent than the placebo [23,31]. This highlights the need to explore new therapeutic approaches for BMS that not only alleviate subjective symptoms but also restore overall health-related and oral health-related quality of life. An attempt was made to develop a model describing the quantitative relationship between the therapeutic effect at the final evaluation and independent clinical and psychological variables. It was demonstrated that the final NRS-11 score may have had a possible influence by sex (greater reduction in burning among women), taste disturbances (greater reduction in the absence of this somatosensory symptom), depression (greater reduction in the absence of depression as measured by the BDI), and age (greater reduction in older patients). These findings should be interpreted with caution, primarily due to the relatively small number of patients assessed; however, during model validation, only the assumption concerning the expected value of the random error term εi being equal to zero was not fully met. In a logistic regression model based on 212 BMS patients treated either systemically or topically with clonazepam, a greater reduction in burning was observed in those with higher baseline subjective pain scores and in patients with circadian rhythm disturbances, whereas the presence of comorbid depression had a negative impact on treatment outcomes [38]. In the same patient group, the application of machine learning for treatment outcome prediction identified 41% of individuals with a good response and 84% with a poor response, based on selected prognostic parameters. Among these, the significant impact of baseline burning intensity was specifically confirmed, and a history of enteropathy and the presence of burning sensations in oral sites beyond the tongue were also identified as relevant factors [39]. These classical multivariate analyses, along with AI-assisted approaches, serve to identify variables associated with poorer treatment prognosis. It appears that BMS cases involving somatosensory disturbances and comorbid depression present the greatest challenge in achieving meaningful pain reduction using currently available therapeutic methods.

The present study has several limitations. In a split-mouth design, the control sites should ideally be BMS-affected areas covered with a patch lacking the active substance.

This would provide a better opportunity to control for the placebo effect and would enable the conduct of double-blind studies. Comparisons of treatment outcomes in different oral sites with distinct anatomical and histological characteristics should also be interpreted with caution. The duration of active topical therapy with low-concentration capsaicin for neuropathic pain may have been too short, and given the very low incidence of adverse effects, it could potentially be extended to at least 3–4 weeks. The number of participants was not adjusted according to the sample size calculator results due to the proof-of-concept nature of the investigation. The analysis of clinical variables should be expanded, for example, by including a neuropathic pain questionnaire and currently discussed risk factors such as sleep quality disturbances or circadian rhythm disorders. Additionally, the study did not assess the impact of treatment on indicators of general and oral health-related quality of life.

## 5. Conclusions

There is a clear need to establish a structured and individualized treatment strategy for Burning Mouth Syndrome (BMS). The etiological heterogeneity of the condition and distinction between at least a peripheral form characterized by small-fiber neuropathy and a central form potentially related to dysfunction in the dopaminergic system should be taken into account. In the present study, after confirming the peripheral origin of the burning sensation, an innovative mucoadhesive patch was applied, which allowed adverse effects to be avoided. A satisfactory reduction in pain symptom intensity was achieved in almost all treated patients, although it does not yet represent complete remission of disease symptoms and numerous limitations of the conducted study are evident. Further high-quality randomized, double blinded studies with control of the placebo effect in larger patient groups with possibly long-term follow-ups are needed to assess new drugs administered topically in the oral cavity, as well as non-pharmacological methods or their combinations to evaluate the efficacy of new locally administered agents for oral use, as well as non-pharmacological approaches and potential combination therapies.

## Figures and Tables

**Table 1:** Correlation coefficient (R) between baseline pain intensity scores and evaluated variables.

Pain Score	Variable	R Values	*p*	Pain Score	Variable	R Values	*p*
NRS-11	Gender	−0.18	NS	SF-MPQ	Gender	−0.17	NS
	Age	0.08	NS		Age	−0.11	NS
	BDI	0.87	<0.000		BDI	0.61	<0.000
	AIS	0.34	NS		AIS	0.21	NS
	DOM-1	−0.81	<0.000		DOM-1	−0.68	<0.000
	DOM-2	−0.71	<0.000		DOM-2	−0.64	<0.000
	DOM-3	−0.51	0.005		DOM-3	−0,53	0.003
	DOM-4	−0.5	0.005		DPM-4	−0.6	<0.000

**Table 2:** The effect of topical treatment for BMS with 0.025 mg/cm^2^ capsaicin on pain intensity scores.

Type of Variables	Pain Intensity Scores and Means	*p*
Dependent	NRS-11 0 vs. NRS-11 2 6.44 ± 1.6 vs. 2.27 ± 1.5	*p* < 0.000
	SF-MPQ 0 vs. SF-MPQ 2 35.93 ± 7.8 vs. 10.34 ± 7.7	*p* < 0.000
Independent	NRS-11 0 vs. NRS-11c 0 6.44 ± 1.6 vs. 6.24 ± 1.6	*p* = 0.61
	SF-MPQ 0 vs. SF-MPQc 0 35.93 ± 7.8 vs. 34.2 ± 8.1	*p* = 0.41
	NRS-11 2 vs. NRS-11c 2 2.27 ± 1.5 vs. 3.86 ± 1.6	*p* = 0.003
	SF-MPQ 2 vs. SF-MPQc 2 10.34 ± 7.7 vs. 22.65 ± 10.8	*p* < 0.000

NRS-11 0 and 2—NRS-11 scores at baseline and 3 months after treatment. NRS-11c 0 and 2—NRS-11 scores for control sites at baseline and 3 months after treatment. SF-MPQ 0 and 2—SF-MPQ scores at baseline and 3 months after treatment. SF-MPQc 0 and 2—SF-MPQ scores for control sites at baseline and 3 months after treatment.

**Table 3:** Analysis of variance (ANOVA) for mean pain intensity scores at baseline (NRS-11_0_, SF-MPQ_0_), at the end of treatment (NRS-11_1_, SF-MPQ_1_), and 3 months later (NRS-11_2_, SF-MPQ_2_).

Pain Scores	Values	F	*p*	Groups	*p* Tukey Test
NRS-11 0 vs. NRS-11 1 vs. NRS-11 2	6.44 ± 1.6 vs. 3.55 ± 1.6 vs. 2.27 ± 1.5	40.92	<0.000	NRS-11 0 vs. 1 NRS-11 1 vs. 2	<0.000 *p* = 0.022
SF-MPQ 0 vs. SF-MPQ1 vs. SF-MPQ 2	35.93 ± 7.8 vs. 16.37 ± 10.4 vs. 10.34 ± 7.7	68.59	<0.000	SF-MPQ 0 vs. 1 SF-MPQ 1 vs. 2	<0.000 *p* = 0.026

**Table 4:** Comparison of reported pain intensity reduction in BMS after topical capsaicin application with the findings of the present study.

Author, Country	N	Age Mean	Topical Form of Capsaicin	Control Group	NRS-11 Baseline	NRS-11 at End Therapy	Follow-Up NRS-11
Marino et al. [12], Italy, 2010	14	ND	Oral rinse	ALA Boric acid	6.1 ± 2.2	8 weeks: 2.9 ± 2.6	2 months: 2.9 ± 3.0
Silvestre et al. [13], Spain, 2012	23	72.6	0.02% oral rinse	Placebo	6.0 ± 3.1	1 week: 3.7	-
Jørgensen et al. [14], 2017 Denmark	18	61	0.01% and 0.025% oral gel	-	6.4 ± 2.0 4.6 ± 2.6	4 weeks: 5.1 ± 3.0 3.0 ± 2.9	2 weeks: 6.0 ± 2.7 3.2 ± 3.1
Ricken et al. [15], Brazil, 2021	18	30–69	0.025% oral gel	-	7.4 ± 2.5	12 weeks 2.1 ± 2.3	4 months: 0.9 ± 1.2
Jankovskis et al. [16], Latvia 2021	20	ND	0.02% oral rinse	Vitamin B, zinc	median 3.0	3 weeks: median 1.5	
Own study	29	61.8	0.025 mg/cm^2^ mucoadhesive polymer-film	Other sites	6.4 ± 1.6	2 weeks: 3.6 ± 1.6	3 months: 2.3 ± 1.5

ND—no data.

## Data Availability

The raw data supporting the conclusions of this article will be made available by the authors upon request.

## References

[1] (2018). Headache Classification Committee of the International Headache Society (IHS) The international classification of headache disorders, 3rd edition. Cephalalgia.

[2] The Orofacial Pain Classification Committee (2020). International classification of orofacial pain, 1st edition (ICOP). Cephalalgia.

[3] Tan H.L., Renton T. (2020). Burning mouth syndrome: An update. Cephalalgia Rep..

[4] Yilmaz Z., Renton T., Yiangou Y., Zakrzewska J., Chessell I.P., Bountra C., Anand P. (2007). Burning mouth syndrome as a trigeminal small fiber neuropathy: Increased heat and capsaicin receptor TRPV1 in nerve fibers correlates with pain score. J. Clin. Neurosci..

[5] Beneng K., Yilmaz Z., Yiangou Y., McParland H., Anand P., Renton T. (2010). Sensory purinergic receptor P2X3 is elevated in burning mouth syndrome. Int. J. Oral Maxillofac. Surg..

[6] Jääskeläinen S.K. (2012). Pathophysiology of primary burning mouth syndrome. Clin. Neurophysiol..

[7] Hagelberg N., Forssell H., Aalto S., Rinne J.O., Scheinin H., Taiminen T., Någren K., Eskola O., Jääskeläinen S.K. (2003). Altered dopamine D2 receptor binding in atypical facial pain. Pain.

[8] Blanchet P.J., Brefel-Courbon C. (2018). Chronic pain and pain processing in Parkinson’s disease. Prog. Neuropsychopharmacol. Biol. Pychiatry.

[9] Nasri-Heir C., Shigdar D., Alnaas D., Korczeniewska O.A., Eliav R., Heir G.M. (2017). Primary burning mouth syndrome: Literature review and preliminary findings suggesting possible association with pain modulation. Quintessence Int..

[10] Tan H.L., Smith J.G., Hoffmann J., Renton T. (2022). A systematic review of treatment for patients with burning mouth syndrome. Cephalalgia.

[11] Petruzzi M., Lauritano D., De Benedittis M., Baldoni M., Serpico R. (2004). Systemic capsaicin for burning mouth syndrome: Short-term results of a pilot study. J. Oral Pathol. Med..

[12] Marino R., Torretta S., Capaccio P., Pignataro L., Spadari F. (2010). Different therapeutic strategies for burning mouth syndrome: Preliminary data. J. Oral Pathol. Med..

[13] Silvestre F.J., Silvestre-Rangil J., Tamarit-Santafé C., Bautista D. (2012). Application of a capsaicin rinse in the treatment of burning mouth syndrome. Med. Oral Patol. Oral Cir. Bucal.

[14] Jørgensen M.R., Pedersen A.M. (2017). Analgesic effect of topical oral capsaicin gel in burning mouth syndrome. Acta Odontol. Scand..

[15] Ricken C.M., Pƒder S.N., Kamikawa D.S., Pieralisi N., Chicarelli M., Tolenetino E.S. (2021). Evaluation of a protocol for topical application of capsaicine gel 0.025% in the management of burning mouth syndrome correlating its impact on quality of life. Int. J. Odontostomat.

[16] Jankovskis V., Selga G. (2021). Vitamin B and zinc supplements and capsaicin oral rinse treatment options for burning mouth syndrome. Medicina.

[17] Arora V., Campbell J.N., Chung M.K. (2021). Fight fire with fire: Neurobiology of capsaicin-induced analgesia for chronic pain. Pharmacol. Ther..

[18] Grémeau-Richard C., Dubray C., Aublet-Cuvelier B., Ughetto S., Woda A. (2010). Effect of lingual nerve block on burning mouth syndrome (stomatodynia): A randomized crossover trial. Pain.

[19] Farrar J.T., Young J.P., LaMoreaux L., Werth J.L., Poole M.R. (2001). Clinical importance of changes in chronic pain intensity measured on an 11-point numerical pain rating scale. Pain.

[20] Melzack R. (1987). The short-form McGill Pain Questionnaire. Pain.

[21] Beck A.T., Steer R.A., Ball R., Ranieri W. (1996). Comparison of Beck Depression Inventories -IA and -II in psychiatric outpatients. J. Pers. Assess..

[22] Soldatos C.R., Dikeos D.G., Paparrigopoulos T.J. (2003). The diagnostic validity of the Athens Insomnia Scale. J. Psychosom. Res..

[23] Skevington S.M., Lotfy M., O’Connell K.A. (2004). The World Health Organization’s WHOQOL-BREF quality of life assessment: Psychometric properties and results of the international field trial. A report from the WHOQOL group. Qual. Life Res..

[24] Dołowacka-Jóźwiak A., Nawrot-Hadzik I., Matkowski A., Ciecieląg T., Gawin-Mikołajewicz A., Dudek-Wicher R., Prochoń M., Markowska D., Adamski R., Wiater A. (2025). Mucoadhesive PVA Film for Sustained Resveratrol Delivery: Formulation, Characterization, and Release Profile. Molecules.

[25] Kuzma M., Fodor K., Boros B., Perjési P. (2015). Development and Validation of an HPLC-DAD Analysis for Pharmacopoeial Qualification of Industrial Capsicum Extracts. J. Chromatogr. Sci..

[26] Kitisomprayoonkul W., Klaphajone J., Kovindha A. (2006). Thai short-form McGill Pain Questionnaire. J. Med. Assoc. Thai..

[27] Koivisto A.P., Voets T., Iadarola M.J., Szallasi A. (2024). Targeting TRP channels for pain relief: A review of current evidence from bench to bedside. Curr. Opin. Pharmacol..

[28] Khemiss M., Chaabouni D., Ben Khaled R., Ben Khélifa M. (2022). Place of placebo therapy in the treatment of burning mouth syndrome: A systematic review. Dent. Med. Probl..

[29] Kuten-Shorrer M., Kelley J.M., Sonis S.T., Treister N.S. (2014). Placebo effect in burning mouth syndrome: A systematic review. Oral Dis..

[30] Rossetti A., Teixeira A., Milhazes N. (2025). Efficacy of different therapeutic options for pain relief and treatment of burning mouth syndrome: A systematic review. Clin. Oral Investig..

[31] Colloca L. (2019). The placebo effect in pain therapies. Annu. Rev. Pharmacol. Toxicol..

[32] Alvarenga-Brant R., Costa F.O., Mattos-Pereira G., Esteves-Lima R., Belém F., Lai H., Ge L., Gomez R., Martins C. (2022). Treatments for burning mouth syndrome: A network meta-analysis. J. Dent. Res..

[33] Derry S., Moore R.A. (2012). Topical capsaicin (low concentration) for chronic neuropathic pain in adults. Cochrane Database Syst. Rev..

[34] Rupel K., Buoite Stella A., Tamos M., Adamo D., Canfora F., Biasotto M., Di Lenarda R., Ottaviani G. (2025). Effects of oral topical capsaicin gel on taste perception in healthy subjects: A pilot study. J. Oral Pathol. Med..

[35] Kim J.l., Kim Y.S., Ko I., Kim D. (2020). Association between burning mouth syndrome and the development of depression, anxiety, dementia, and Parkinson disease. JAMA Otolaryngol. Head Neck Surg..

[36] Freilich J.E., Kuten-Shorrer M., Treister N.S., Woo S.B., Villa A. (2020). Burning mouth syndrome: A diagnostic challenge. Oral Surg. Oral Med. Oral Pathol. Oral Radiol..

[37] Alhendi F., Ko E., Graham L., Corby P. (2023). The association of sleep disturbances with burning mouth syndrome: An overlooked relationship—A qualitative systematic review. Oral Dis..

[38] Kim M.J., Kim J., Kho H.S. (2021). Treatment outcomes and related clinical characteristics in patients with burning mouth syndrome. Oral Dis..

[39] Kim M.J., Kim P.J., Kim H.G., Kho H.S. (2021). Prediction of treatment outcome in burning mouth syndrome patients using machine learning based on clinical data. Sci. Rep..

